# Examination of predictable factors of perioperative respiratory complications by preoperative forced oscillation technique parameters

**DOI:** 10.17179/excli2020-3087

**Published:** 2020-12-21

**Authors:** Akira Igarashi, Sumito Inoue, Yoko Shibata, Keiko Nunomiya, Takahito Ota, Yu Ishibashi, Hiroaki Murano, Kodai Furuyam, Sujeong Yang, Hiroyoshi Machida, Hiroshi Nakano, Kento Sato, Masamichi Sato, Takako Nemoto, Michiko Nishiwaki, Keiko Yamauchi, Jun Suzuki, Mitsuaki Sadahiro, Masafumi Watanabe

**Affiliations:** 1 Department of Cardiology, Pulmonology, and Nephrology, Yamagata University Faculty of Medicine, Yamagata, Japan; 2 Department of Pulmonary Medicine, Fukushima Medical University, Fukushima, Japan; 3 Second Department of Surgery, Yamagata University Faculty of Medicine, Yamagata, Japan

**Keywords:** forced oscillation technique parameter, general anesthesia, perioperative respiratory complications, pulmonary function test, surgery

## Abstract

In this study, we investigated whether pulmonary function tests such as forced oscillation technique parameters could predict perioperative respiratory complications. In the results of our study, perioperative respiratory complications cannot be predicted using the results of preoperative pulmonary function tests and forced oscillation technique parameters. Patients who are judged by comprehensive preoperative judgment to be suitable for general anesthesia may not need to consider the risk of perioperative complications using pulmonary function test.

## ⁯

***Dear Editor,***

In surgery with general anesthesia, it is important to predict the occurrence of perioperative respiratory complications (Gass and Olsen, 1986[4]). Previously, to predict the onset of perioperative respiratory complications, their relevance to preoperative pulmonary function tests has been examined (Bolliger and Perruchoud, 1998[3]). However, there is not enough evidence to support the use of pulmonary function tests, and the results of pulmonary function tests cannot predict the occurrence of perioperative respiratory complications (Almquist et al., 2018[2].). To evaluate respiratory status, the forced oscillation technique (FOT) is used as a simple method as opposed to standard methods of measuring pulmonary function, such as forced vital capacity (FVC) and forced expiratory volume in one second (FEV_1_). MostGraph (Chest M.I. Inc., Tokyo Japan) is a device that measures the resistance and reactance of the respiratory system using the FOT and was developed in Japan (Ohishi and Kurosawa, 2011[5]). Airway resistance and reactance can be measured quickly under normal tidal volume and the results displayed in three dimensions using MostGraph (Abe et al., 2016[1].). Parameters of the FOT include Rrs and Xrs. Rrs is defined as the total resistance of the respiratory system, while Xrs reflects the sum of the elastic and inertial properties of the respiratory system. Rrs and Xrs at 5 Hz are defined as R5 and X5, respectively. Rrs at 20 Hz is defined as R20, and the difference between Rrs at 5 Hz and 20 Hz is depicted by: R5 − R20. The resonance frequency at which Xrs = 0 is defined as Fres, and the low-frequency reactance area is defined as ALX. Respiratory resistance R5 − R20 is associated with indicators of frequency dependence of resistance, which is presumed to reflect ventilation inhomogeneity. Respiratory reactance X5 with peripheral airway lesions is significantly associated with chronic obstructive pulmonary disease (COPD) dyspnea score (Shirai and Kurosawa, 2016[6]). The value of R5 reflected total respiratory system resistance, and the value of X5 reflected total respiratory system reactance (Stevenson et al., 2005[7]). 

In this study, we enrolled 103 patients who underwent surgery under general anesthesia from January to December 2015 (Table 1(Tab. 1)). The Institutional Ethics Committee of Yamagata University Faculty of Medicine approved all procedures (approval number: H26-36; approval date: 16^th^ June, 2014). Written informed consent was obtained from all subjects. This study was performed according to the principles outlined in the Declaration of Helsinki. Fifty-five patients underwent cardiovascular surgery, and 48 patients underwent lung or mediastinal surgery. There were 25 cases of perioperative respiratory complications (7 cases of respiratory tract infection, 7 cases of pleural fluid, 4 cases of respiratory failure, 4 cases of atelectasis, and 3 cases of pulmonary fistula [with partial duplication]). No deaths due to perioperative respiratory complications were observed. There were no significant differences in FVC or FEV_1_ between groups with or without perioperative respiratory complications. There was also no significant difference between groups in FOT parameters (Table 1(Tab. 1)).

In this study, in patients who underwent thoracic surgery with general anesthesia, preoperative pulmonary function tests and FOT parameters did not predict perioperative respiratory complications. Patients who are deemed suitable to undergo general anesthesia by comprehensive preoperative judgment may not need to consider the risk of perioperative complications using pulmonary function tests.

## Acknowledgement

The authors would like to thank Enago (www.enago.jp) for the English language review.

## Authors’ contributions

Akira Igarashi: Data curation, Original draft preparation, Sumito Inoue: Conceptualization, Study design, Yoko Shibata: Conceptualization, Writing-Reviewing, Keiko Nunomiya: Administration of data base, Takahito Ota, Yu Ishibashi, Hiroaki Murano, Kodai Furuyama, Sujeong Yang, Hiroyoshi Machida: Data analysis. Hiroshi Nakano, Kento Sato, Masamichi Sato, Takako Nemoto, Michiko Nishiwaki, Keiko Yamauchi, Jun Suzuki: Data collection, Explanation and receipt of informed consent from patients, Mitsuaki Sadahiro, Masafumi Watanabe: Reviewing and Editing, All authors: Data interpretation, Final approval of the manuscript.

## Funding disclosure

This work was supported by JSPS KAKENHI Grant Number JP26861222.

## Declaration of competing interest

AI received lecture fees from AstraZeneca K.K., Boehringer Ingelheim Japan.

SI received lecture fees from Otsuka Pharmaceutical Co., Ltd, Novartis Pharma K.K., AstraZeneca K.K., GlaxoSmithKline K.K., Chugai Pharmaceutical Co., Ltd., Boehringer Ingelheim Japan, MSD K.K., ONO PHARMACEUTICAL CO., LTD., Meiji Seika Pharma Co., Ltd., KYORIN Pharmaceutical Co.,Ltd., Taiho Pharmaceutical Co.,Ltd., Eli Lilly Japan K.K., NIHON PHARMACEUTICAL CO., LTD., TEIJIN PHARMA LIMITED., Pfizer Japan Inc., DAIICHI SANKYO COMPANY, LIMITED, and received research grant from Chugai Pharmaceutical Co., Ltd., Novartis Pharma K.K., MSD K.K., KYORIN Pharmaceutical Co.,Ltd., and JSPS KAKENHI (grant number 19K08658).

YS received lecture fees from Boehringer Ingelheim Japan, AstraZeneca K.K., Novartis Pharma K.K., KYORIN Pharmaceutical Co.,Ltd., Chugai Pharmaceutical Co., Ltd., Pfizer Japan Inc., GlaxoSmithKline K.K., CHEST, received advisory fee from GlaxoSmithKline K.K., and received research grant from Boehringer Ingelheim Japan, AstraZeneca K.K., Novartis Pharma K.K., KYORIN Pharmaceutical Co.,Ltd., Chugai Pharmaceutical Co., Ltd., and JSPS KAKENHI (grant number 19K08658). 

TO received lecture fee from MSD K.K.

KF received lecture fee from Boehringer Ingelheim Japan.

SY received lecture fees from Boehringer Ingelheim Japan, AstraZeneca K.K. 

HMa received lecture fees from Boehringer Ingelheim Japan.

HN received lecture fees from Pfizer Japan Inc., Boehringer Ingelheim Japan, AstraZeneca K.K. 

KS received lecture fee from Chugai Pharmaceutical Co., Ltd., GlaxoSmithKline K.K., AstraZeneca K.K., Sanofi K. K. and research grant from GlaxoSmithKline K.K.

MS received lecture fees from AstraZeneca K.K., Boehringer Ingelheim Japan. 

TN received lecture fee from Teijin Pharma Ltd Japan. 

KY received lecture fee from Pfizer Japan Inc. 

MW received research grants from MSD K.K., Asahi Kasei Pharma Corporation., Asahi Kasei Medical Co., Ltd., KISSEI PHARMACEUTICAL CO., LTD., Kyowa Kirin Co.,Ltd., Sanofi K.K., SHIONOGI & CO., LTD., Taisho Pharma Co., Ltd., CHUGAI PHARMACEUTICAL CO., LTD., TEIJIN PHARMA LIMITED., Torii Pharmaceutical Co., Ltd., Nihon Medi-Physics Co.,Ltd., Novartis Pharma K.K., BIOTRONIK Japan, Inc., FUJIFILM Toyama Chemical Co., Ltd., Roche Diagnostics K.K., Kowa Company, Limited, Abbott Vascular Japan Co., Ltd., Cardinal Health Japan, Kowa Company, Limited, research grants and personal fees from Actelion Pharmaceuticals Japan Ltd., Astellas Pharma Inc., Otsuka Pharmaceutical Co., Ltd., ONO PHARMACEUTICAL CO., LTD., DAIICHI SANKYO COMPANY, LIMITED, Sumitomo Dainippon Pharma Co., Ltd., Takeda Pharmaceutical Company Limited, Nippon Boehringer Ingelheim Co ., Ltd., MEDTRONIC JAPAN CO., LTD., Bayer Yakuhin, Ltd., Pfizer Japan Inc., MOCHIDA PHARMACEUTICAL CO.,LTD., personal fees from Mitsubishi Tanabe Pharma Corporation, KOWA PHARMACEUTICAL COMPANY LTD., TOA EIYO LTD., Bristol-Myers Squibb K.K., AstraZeneca K.K., Edwards Lifesciences Corporation, Amgen. Inc., Japan Lifeline Co., Ltd..

KN, YI, HMu, MN, JS, and MS have no COI.

## Figures and Tables

**Table 1:**
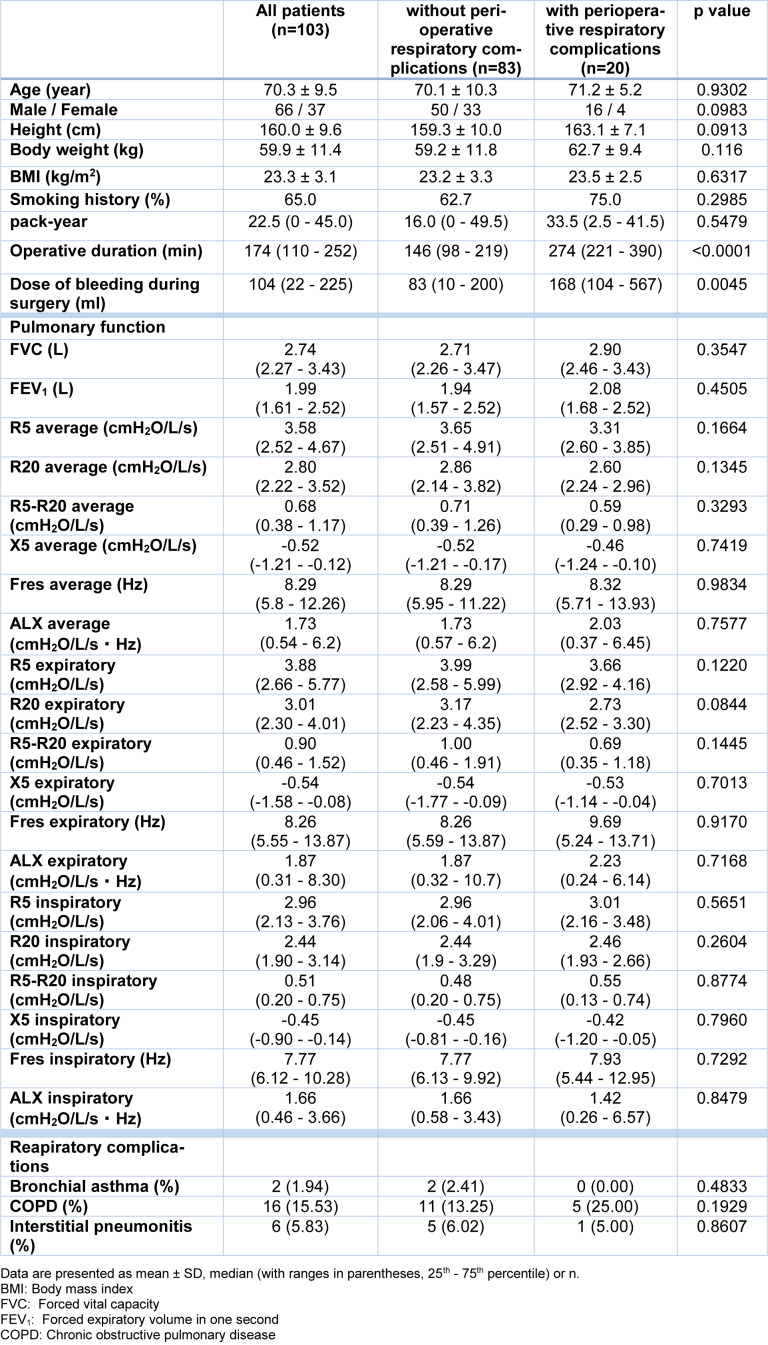
Comparison of clinical characteristics and data of pulmonary function between subjects with or without perioperative respiratory complications.
